# Intrahepatic Hemorrhage of Traumatic Origin; Operation: Recovery

**Published:** 1912-09

**Authors:** Gaston Torrance

**Affiliations:** Birmingham, Ala.; Surgeon to St. Vincent’s Hospital


### INTRAHEPATIC HEMORRHAGE OF TRAUMATIC ORIGIN; OPERATION; RECOVERY.

By Gaston Torrance, M. D., Birmingham, Ala.

*Surgeon to St. Vincent's Hospital.*

A man twenty-five years of age was brought to me, with a history of having been injured twelve days previously. At the time of the injury he was standing in the mines by a post near the car track, when a trip of cars jumped the track and one of them struck him in the back over the region of the left kidney; the impact of the car threw him against the post, which struck him to the right of the median line.

For the first four of five days he suffered pain over the kidney and passed some bloody urine; he then began to have pain over the line radiating around the margins of the ribs and noticed an enlargement in the region of the gall bladder. He had no rise of temperature and was not confined to bed all of the time.

When admitted to the hospital there was a decided enlargement over the gall-bladder with some tenderness and slight rigidity, there was a slight tinge of yellow, his temperature was normal, white blood count 10,500, the urine showed a trace of albumen, some pus and red blood cells.

I concluded that we had a very much distended gall-bladder and decided to operate at once. On opening the abdomen the gall-bladder was found to be normal, but there was a considerable enlargement of the liver between the gall-bladder and the median line, which seemed to be nearer the surface on the under surface of the liver.

With this history of injury we decided that we were dealing with an intrahepatic hemorrhage. As the enlargement was so gradual and so recent, there was some question as to whether the bleeding had ceased or not. To leave it was to invite infection and abscess formation.

After thoroughly protecting the abdominal cavity with gauze packs, a trocar was passed into this bulging part of the under surface of the liver and about a pint of fluid blood was drawn off, the opening was then enlarged enough to admit two fingers for exploration of the cavity. A rubber tube wrapped with gauze and rubber dam was introduced and a gauze drain covered with rubber dam was placed beneath the liver at this point and the omentum was then packed around the drains.

He was in good condition at the end of the operation and was placed in bed with the foot elevated. He made an uneventful recovery.

A few days after operation there was a free flow of bile, which continued for about two weeks.

For the first week he was kept on a very limited diet, when we began to give him a more liberal diet, but found this made him quite ill with nausea, etc., probably due to the destruction of liver substance.

He left the hospital at the end of the third week with only a small sinus draining mucus and was gradually increasing his diet.

---

### NEIGHBORHOOD LIBRARY ASSOCIATION.

The Neighborhood Library Association, 227 S. Humphries St., Atlanta, Ga., establishes free libraries of popular and standard books in needy neighborhoods, country settlements, mountain districts, and poor schools. There is absolutely no charge to anyone. It is purely a philanthropic, public-spirited, and charitable work, and depends solely upon voluntary contributions of money and donations of books sent in by those who realize the great need and who are able and willing to help, even a little, in this good cause of general education and intellectual enlightenment. The work was started by a Baptist Minister who is still President of the Association, but it is strictly undenominational. It is not for the dissemination of sectarian views, but for the encouragement of general reading, mostly fiction and history.

Communications are requested from those who want libraries located in their nighborhood and also from those who will donate either money or books.

---

*To the Readers of the Journal-Record of Medicine*:

About six years ago the writer began to use vaccines in the treatment of typhoid fever. Since that time he has thus treated more than one hundred cases and has obtained numerous articles upon the same subject written by physicians in various parts of the world. It seems possible, however, that some may have escaped notice. He also realizes that many of the profession may have treated some cases without reporting them. A paper upon the subject is now in the course of preparation. In this it is earnestly desired to incorporate reports from a large number of cases, good, bad, or otherwise. He accordingly makes the following request to the readers of this journal:

Will any one who has used vaccines in the treatment of typhoid fever, whether but one case or more, kindly communicate to him that fact, accompanied by name and address of the reporter. If the results have already been reported, a note of the journal in which they appeared will be sufficient. If they have not been reported, a short blank form will be sent to the physician to be filled out. Due credit will be given in the article to each person making a report. If any physician happens to know of other confreres who have any such cases, it will be appreciated if he sends their names, as they may not happen to read this note. It is hoped that by this means a sufficient number of cases may be collected to somewhat definitely settle the now mooted question whether vaccines are or are not of benefit in typhoid therapy.

Reports of cases will be accepted at any time in the future, but preferably by November or December of the present year.

Kindly communicate with Dr. W. H. Watters, Director of the Department of Pathology and Bacteriology, Evans Institute for Clinical Research, Boston, Mass.

